# Variante no descrita previamente en el gen *COL3A1*: causalidad del Síndrome de Ehlers-Danlos tipo Vascular

**DOI:** 10.1515/almed-2025-0096

**Published:** 2025-08-12

**Authors:** Estrella Gutiérrez Romero, Nuria Padilla Apuntate, Silvia Izquierdo Álvarez

**Affiliations:** 16488 Servicio de Bioquímica Clínica del Hospital Universitario Miguel Servet, Zaragoza, España

**Keywords:** *COL3A1*, fragilidad, ictus, Síndrome de Ehlers-Danlos de tipo Vascular

## Abstract

**Objetivos:**

Diagnosticar a través del estudio genético el Síndrome de Ehlers-Danlos de tipo Vascular (SEDV), caracterizado por roturas musculares y arteriales, así como tendencia a la formación de hematomas, piel fina con venas visibles y rasgos faciales acrogénicos y cuya causalidad es debida a variantes patogénicas (VP) en heterocigosis en el gen *COL3A1*.

**Caso clínico:**

Presentamos el caso de un varón que, tras sufrir un ictus, se identifica una variante probablemente patogénica no reportada previamente en el gen *COL3A1*, que cambió la sospecha inicial de Síndrome de Marfan por el diagnóstico genético definitivo de SEDV.

**Conclusiones:**

La variante detectada en el gen *COL3A1* podría apoyar la causalidad en el contexto clínico el paciente y proporcionar un mejor manejo terapéutico.

## Introducción

El Síndrome de Ehlers-Danlos de tipo Vascular (SEDV), se caracteriza por rotura arterial e intestinal, la rotura uterina durante el embarazo, así como tendencia a la formación de hematomas, piel fina con venas visibles y rasgos faciales acrogéricos. Todo ello provocado por una mutación heterocigota en el gen *COL3A1,* patología que se hereda de manera autosómica dominante.

## Caso clínico

**Figura 1:**
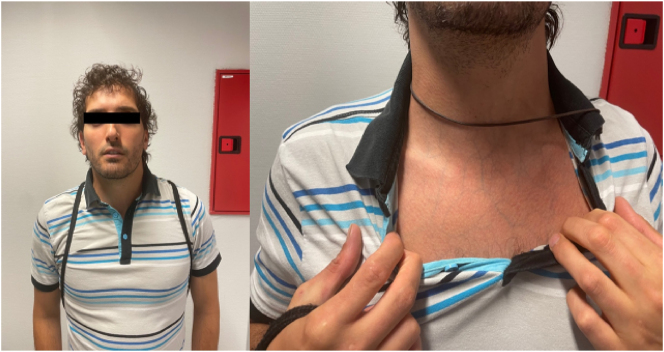
Rasgos faciales acrogéricos: nariz puntiaguda, labio superior fino, ojos prominentes, mejillas hundidas. Venas visibles, piel fina y traslúcida.

Paciente varón de 35 años, con antecedentes personales de fragilidad muscular con múltiples roturas a nivel de: bíceps, pectoral mayor y pene (hasta en tres ocasiones). Fenotipo del paciente: rasgos faciales acrogéricos y piel fina y traslúcida (Figura 1).

Antecedente familiar, muerte idiopática de su padre tras una broncoscopia.

En enero de 2025 durante un partido de waterpolo presentó un cuadro de dolor cervical derecho irradiado al ojo ipsilateral, mareo y amaurosis en ojo derecho. Posteriormente, somnolencia, disartria y debilidad en extremidades izquierdas, por lo que fue trasladado al Hospital.

El paciente refirió que, días previos, estuvo trabajando en la construcción de una casa, adoptando posturas forzadas del cuello y levantando cargas pesadas. El día del episodio, aseguró malestar hemicraneal derecho que, posteriormente identificó como el mismo dolor. Lo atribuyó inicialmente al gorro de natación y lo aflojó. Tras realizar un lanzamiento, notó pérdida de visión en el ojo derecho.

A la exploración: hipotenso (104/59 mmHg), somnoliento, disartria moderada, hemianopsia homónima izquierda, anisocoria derecha con discreta ptosis, parálisis facial central izquierda severa, hemiplejía braquiocrural izquierda asociada a hemianestesia y puntuación de 20 en National Institutes of Health Stroke Scale.

En tomografía computarizada se evidenció disección carotídea derecha con trombo en arteria cerebral media (ACM). Ante la sospecha de un Ictus isquémico agudo (Infarto Total de la Circulación Anterior) derecho secundario a disección carotídea con trombo en M1 de la ACM derecha, se inició fibrinólisis intravenosa. Se derivó para trombectomía, pero al no evidenciarse oclusión completa del vaso y haber recanalización completa de la ACM, se detuvo el procedimiento por alto riesgo de embolismo.

A nivel analítico destacaron niveles ligeramente elevados de triglicéridos y de proteína C reactiva (PCR) (Tabla 1).

**Tabla 1:** Resultados de la analítica realizada al paciente.

Parámetro	Valor	Unidades	Rango referencia
NT-proBNP en suero	32	pg/mL	≤103
TSH en suero	1,68	μUl/mL	0,38–5,33
PCR	1,25^a^	mg/dL	≤0,50
Urea en suero	30	mg/dL	17–43
Creatinina en suero	0,78	mg/dL	0,67–1,17
Triglicéridos en suero	194^a^	mg/dL	30–175
Colesterol en suero	209	mg/dL	120–220
Bilirrubina total en suero	0,47	mg/dL	0,30–1,20
Proteínas totales en suero	7	g/dL	6,60–8,30
GFR-CKD EPI	116,95	mL/min	≥90
FA en suero	47	U/L	30–120
GGT en suero	36	U/L	≤55
GOT en suero	19	U/L	≤50

TSH, hormona estimulante del tiroides; PCR, proteína C reactiva; GFR-CKD EPI, *glomerular filtration rate-chronic kidney disease epidemiology collaboration* (tasa de filtración glomerular-colaboración epidemiológica sobre la enfermedad renal crónica); FA, fosfatasa alcalina; GGT, gamma-glutamil transferasa; GOT; transaminasa glutámico oxalacética; ^a^indica valor alarma.

El paciente evolucionó favorablemente en 24 h y se decidió alta domiciliaria. Se realizó extracción sanguínea para estudio genético de exoma de conectivopatías y se inició tratamiento con estatinas y adiro.

Desde el laboratorio posteriormente se solicitó el estudio genético mediante Next Generation Sequencing (NGS), exoma clínico dirigido, realizado en LABGENETICS, S.L., estudiando la región codificante de 138 genes (Tabla 2), estudiando la región codificante de 138 genes, así como regiones intrónicas adyacentes (5 pb), para la detección de variantes puntuales y variaciones en el número de copias (CNVs) que pudieran estar asociados al Síndrome de Marfan, hiperlaxitud, aortopatías, Ehlers-Danlos y trastornos relacionados.

**Tabla 2:** Genes estudiados en el Exoma dirigido realizado al paciente.

Genes estudiados asociados al Síndrome de Marfan, Hiperlaxitud articular, Aortopatías, Ehlers Danlos y trastornos relacionados (138 genes)
*ABCC6, ABCC9, ABL1, ACTA2, ADAMTS10, ADAMTS17, ADAMTS2, ADAMTSL2, ADAMTSL4, AEBP1, ALDH18A1, ATP6V0A2, ATP7A, B3GALT6, B3GAT3, B4GALT7, BGN, BMP1, BPNT2, BRAF, C1R, C1S, CANT1, CBS, CHST14, CHST3, COL11A1, COL11A2, COL12A1, COL1A1, COL1A2, COL2A1, COL3A1, COL4A5, COL5A1, COL5A2, COL6A1, COL6A2, COL6A3, COL9A1, COL9A2, COL9A3, CRTAP, CST3, DLG4, DSE, EFEMP2, ELN, ENPP1, EXOC6B, FBLN5, FBN1, FBN2, FGD1, FGFR3, FKBP10, FKBP14, FLNA, FLNB, FMR1, FOXE3, GAA, GALNS, GATA5, GGCX, GORAB, HCN4, HRAS, IFITM5, KCNJ8, KDM6A, KIF22, KMT2D, KRAS, LOX, LTBP2, LTBP3, LTBP4, LZTR1, LZTS1, MAP2K1, MAP2K2, MAT2A, MED12, MFAP5, MSTN, MYH11, MYH7. MYLK, MYLK2, NKX2-5, NOTCH1, NOTCH3, NRAS, P3H1, PLOD1, PLOD2, PLOD3, PLP1, PPIB, PRDM5, PRKG1, PTPN11, PYCR1, RAF1, RIN2, RIT1, ROBO3, RYR1, SERPINH1, SKI, SLC26A2, SLC2A10, SLC39A13, SMAD2, SMAD3, SMAD4, TTN, SMS, SOS1, SOS2, SP7, SSPN, TAB2, TGFB2, TGFB3, TGFBR1, TGFBR2, TNFRSF1A, TNXB, TRPS1, TTN, TYMP, UPF3B, VCAN, XYLT1, ZDHHC9, ZNF469*

Se utilizó el Exome Panel v2.5 (Illumina), para la preparación de librerías y enriquecimiento mediante sondas de captura, que cubre la región codificante (± 5 pb regiones splicing) de 19.396 genes, con una cobertura (on-target) de más del 97 % a 10×, para la detección de variantes puntuales (SNVs e Indels) y CNVs (grandes deleciones/duplicaciones). Secuenciación con la plataforma Illumina NextSeq^®^2000 (Blackhills Diagnostic Resources (BDR), Zaragoza, España). Análisis bioinformático y alineamiento de las secuencias obtenidas frente al genoma de referencia hg19 mediante el software Dragen v4.3 (Illumina). Análisis e interpretación de las variantes mediante Illumina Emedgene, ACMG score, Alamut^®^ Visual 1.9, gnomAD browser, Varsome y ClinVar. Validación de las variantes informadas mediante secuenciación Sanger.

En dicho estudio se detectó una variante probablemente patogénica (VPP), en el gen *COL3A1*, la c.1977+1G>C, p.(?), que podría afectar al sitio donador de splicing (5′-donor splice site) del intrón 28 del gen *COL3A1*, lo que podría introducir un fallo en el *splicing* de la proteína, que tendría como consecuencia un *splicing* aberrante en el mRNA de la proteína codificada por este gen, y haría que ésta no fuese funcional.

Esta variante no había sido descrita previamente como una variante patogénica (VP) asociada al desarrollo de ninguna patología, ni está incluida en ninguna de las bases de datos consultadas (LOVD, ClinVar, dbSNP, gnomAD), por lo que se trata de una variante no descrita con anterioridad.

De acuerdo con los criterios actualizados de American College of Medical Genetics (ACMG), la variante se clasificó como VPP (PVS1, PM2), y puesto que VP en el gen *COL3A1* están asociadas al desarrollo del Síndrome de Ehlers-Danlos de tipo Vascular (OMIM#130050), su presencia en heterocigosis podría ser diagnóstica para el desarrollo de esta patología y apoyaría la causalidad del cuadro clínico [1].

No se detectó ninguna variante descrita como VP o VPP, en los 81 genes que la ACMG (v3.2) recomienda reportar en caso de hallazgos secundarios [2].

Puesto que el Síndrome de Ehlers-Danlos (SED) por VP en el gen *COL3A1* presenta un patrón de herencia AD, se recomendó realizar el estudio de dicha variante en familiares directos del paciente.

## Discusión

El diagnóstico inicial de sospecha se basa en antecedentes familiares o personales de disecciones arteriales, roturas o aneurismas o complicaciones en el embarazo a edades tempranas. El 50 % de los pacientes pueden tener mutaciones *de novo* [3].

El SEDV comprende alrededor del 4–5 % de los casos de SED y tiene una alta tasa de complicaciones fatales relacionadas con sistemas cardiovascular y gastrointestinal, como hemorragia cerebrovascular o rotura intestinal [4].

Aunque las roturas musculares se incluyen en los criterios diagnósticos menores del SEDV, la afectación musculoesquelética exclusiva no es una presentación clínica común de la enfermedad. Por lo tanto, en ausencia de antecedentes familiares, el diagnóstico del SEDV puede ser difícil en un paciente que solo presenta roturas musculares, incluso si son múltiples [5].

Ante este fenotipo, el síndrome de Marfan y la colagenopatía deben incluirse en el diagnóstico diferencial, ya que comparten características clínicas similares. De hecho, tras descartar el síndrome de Marfan mediante la evaluación de la Escala Sistémica de Marfan o el análisis de *FBN1*, los clínicos deben considerar la colagenopatía en sus diferentes formas, posiblemente atípicas [6].

El diagnóstico de SEDV requiere confirmación molecular para diferenciarlo de afecciones con una presentación similar, este se confirma al encontrar una VP en un alelo del gen *COL3A1* [7].

Los criterios diagnósticos del SEDV deben considerarse en individuos con un criterio mayor o varios criterios menores. Mayores: aneurismas arteriales, disecciones o roturas, rotura intestinal, rotura uterina durante el embarazo y/o historia familiar de SEDV. Menores: neumotórax, facilidad para formación de hematomas, piel fina y traslúcida, pie equinovaro, rasgos faciales acrogéricos, rotura muscular o tendón, venas varicosas a edades tempranas, subluxaciones, entre otros [8].

El espectro clínico se explica, por la heterogeneidad alélica. Las manifestaciones clínicas más comunes son: el neumotórax espontáneo (12 %), la disección de la arteria coronaria y, como consecuencia, infarto de miocardio, rotura espontánea del colon sigmoide, insuficiencia venosa superficial (37 %), fístula carótido cavernosa, protusión ocular y queratocono, fragilidad de las encías (sangrado tras cepillado o uso de hilo dental), así como hiperlaxitud de la articulación temporomandibular (subluxaciones repetidas) y disección de la arteria renal que puede conducir a una disminución del flujo sanguíneo renal, pérdida del parénquima renal e hipertensión renal [9].

Se han identificado en la literatura más de 1.500 variantes del gen *COL3A1* que dan lugar a un fenotipo patógeno. La mayoría de las VP identificadas resultan en sustituciones de un solo aminoácido por glicinas en las repeticiones Gly-XY de la región de triple hélice de la molécula de procolágeno tipo III [10].

La supervivencia media es de unos 51 años y depende de la naturaleza de la VP; siendo más larga en aquellos que presentan una variante nula [11].

El objetivo principal de la intervención médica consiste en mantener la presión arterial en rangos normales o bajos y prevenir picos de presión arterial para minimizar la probabilidad de disección o rotura arterial. Se sugieren medicamentos como: diuréticos, β-bloqueantes, bloqueadores del procesamiento de la angiotensina, y otros agentes antihipertensivos [12].

## Conclusiones

Se reporta una nueva VPP, en el gen *COL3A1* como causa de SEDV, no informada en la literatura científica hasta el momento. Para lograr un diagnóstico adecuado, se deben evaluar los hallazgos clínicos, siendo fundamental el estudio genético en los casos en los que se detectan alteraciones, lo cual tiene implicaciones en el tratamiento y el pronóstico de los pacientes, ya que las estrategias terapéuticas tempranas contribuyen a disminuir la gravedad de los síntomas. El papel del laboratorio y la realización del estudio genético fue clave para el diagnóstico del paciente, y poder así ofrecer asesoramiento genético y reproductivo, tanto al paciente como a los familiares directos.

## Supplementary Material

Supplementary Material

## Material Suplementario

Este artículo incluye material suplementario (https://doi.org/10.1515/almed-2025-0096).
