# Supplementary material for: Variante no descrita previamente en el gen COL3A1: causalidad del Síndrome de Ehlers-Danlos tipo Vascular
Source: Adv Lab Med. 2025 Aug 12;6(4):504–7. [Article in Spanish] doi: 10.1515/almed-2025-0096 (PMC12744372; doi:10.1515/almed-2025-0096)
Supplement: Supplementary file 1 — Supplementary Material [file j_almed-2025-0096_suppl_001.pdf]

USO DE MATERIAL GRÁFICO O/Y AUDIOVISUAL

1.- Objetivos y beneficios que se espera alcanzar

La obtención del material gráfico para documentar aspectos de relevancia clínica de su caso o del de su representado puede ayudar al diagnóstico, seguimiento y tratamiento de su problema médico y al de otros pacientes similares. Su uso con fines científicos, docentes o divulgativos es una práctica habitual que contribuye a mejorar el conocimiento en las ciencias de la salud.

2.- Consecuencias previsibles de su autorización

1. La firma de esta **autorización** es **voluntaria**. Su firma para fines científicos, docentes o divulgativos no condiciona el tratamiento que recibirá usted o su representado, ni pago o ningún otro tipo de beneficio personal para usted. Nunca se utilizarán con fines de publicidad comercial.
2. Si consiente, **se anonimizarán** las fotografías o vídeos tomados que puedan tener utilidad científica y **podrán ser utilizados en publicaciones sanitarias** electrónicas o impresas para **fines docentes o científicos**.
Para ello se eliminará su información personal, como nombre o apellidos y se utilizarán los medios de anonimización necesarios en la imagen o el vídeo, sin perjudicar la utilidad de los mismos (p.ej., tapando la cara o parte de la cara, como los ojos clásicamente). Aun así, es posible que alguien pueda reconocerle a usted o a su representado.
3. Las imágenes o vídeos podrán ser compartidas con fines científicos con miembros del equipo médico-asistencial para fines relacionados con el diagnóstico o tratamiento del paciente concreto.
4. La **negación** de este consentimiento con fines científicos o docentes **no afectará de ninguna manera la atención médica** que se le proporcione a usted o a su tutelado.
5. Podrá retirar este permiso en cualquier momento.
6. Tras la firma del consentimiento también podrá solicitar más información y posteriormente también podrá retirar este permiso en cualquier momento si lo desea.

salud servicio aragonés de salud

Consentimiento Informado

USO DE MATERIAL GRÁFICO O/Y AUDIOVISUAL

DECLARACIONES Y FIRMAS

Etiqueta

Don/doña: DE DIEGO ROYO, RAUL
Fecha de nacimiento: M 35a CGNRL CE
Nº Historia: 628825 AR900031771N
Afiliación: 13926276 13/01/25
Domicilio:
Teléfono:

13926276

Declaro:

Que: Estrella Gutiérrez Romero (nombre y apellidos del profesional que proporciona la información) me ha informado de la necesidad/conveniencia de obtener **material gráfico o/y audiovisual** de mi caso médico. Se me ha explicado y he aceptado y comprendido la información que se me ha dado. El profesional que me ha atendido es quien me ha facilitado las explicaciones en lenguaje claro y sencillo.

He podido formular todas las preguntas que he creído conveniente y me han aclarado las dudas planteadas. he recibido copia de este documento.

Mi aceptación es voluntaria y puedo retirar este consentimiento cuando lo crea oportuno.

En consecuencia, **doy mi consentimiento** para que se obtenga material gráfico o/y audiovisual de mi caso médico con fines:

☒ Científicos ☒ Docentes

En Zaragoza, a 3 de Junio de 2025

Firma del paciente: Raúl de Diego

Firma del profesional: [firma]

Dr./Dra. Estrella Gutiérrez Romero

TUTOR LEGAL O FAMILIAR

☐ **Para menores, confirmación padres no separados**

D./Dña. .... con DNI. ....,
y en calidad de ...., soy consciente de que el paciente cuyos datos figuran en el encabezamiento, no es competente para decidir en este momento, por lo que asumo la responsabilidad de la decisión.

Doy expresamente mi consentimiento para su realización, que podré retirar en el momento que lo desee.

En Zaragoza, a .... de .... de ....

Firma del tutor o representante legal: ....

NO AUTORIZACIÓN / ANULACIÓN

Por la presente **NO AUTORIZO / ANULO** cualquier consentimiento plasmado en el presente impreso, que queda sin efecto a partir de este momento. Entiendo que las imágenes no se emplearán a partir de ahora pero no se pueden retirar las ya utilizadas.

En Zaragoza, a .... de .... de ....

Firma del paciente o representante legal: ....

Información básica sobre protección de datos en cumplimiento del deber de información dispuesto en el RGPD 2016/679:
Responsable: Servicio Aragonés de Salud
Finalidad: Gestión de Consentimientos Informados de Historia Clínica del Servicio Aragonés de Salud
Legitimación: Ley 41/2002 de Autonomía del Paciente, LOPD, RGPD
Destinatarios: No se cederán datos a terceros, salvo obligación legal
Derechos: Acceder, rectificar y suprimir los datos, así como otros derechos, como se explica en la información adicional
Información adicional: Puede consultar información adicional y detallada sobre Protección de Datos en nuestra web:
www.aragon.es/-/proteccion-de-datos-1

SECTOR SANITARIO ZARAGOZA II
